# Possibility of information encoding/decoding using the memory effect in fractional-order capacitive devices

**DOI:** 10.1038/s41598-021-92568-3

**Published:** 2021-06-25

**Authors:** Anis Allagui, Ahmed S. Elwakil

**Affiliations:** 1 Department of Sustainable and Renewable Energy Engineering, University of Sharjah, PO Box 27272 Sharjah, United Arab Emirates; 2 Research Institute of Sciences and Engineering, University of Sharjah, PO Box 27272 Sharjah, United Arab Emirates; 3 Department of Electrical Engineering, University of Sharjah, PO Box 27272 Sharjah, United Arab Emirates; 4 Department of Mechanical and Materials Engineering, Florida International University, Miami, FL 33174 USA; 5 Nanoelectronics Integrated Systems Center, Nile University, Cairo, 12588 Egypt; 6 Department of Electrical and Computer Engineering, University of Calgary, Calgary, Alberta T2N 1N4 Canada

**Keywords:** Supercapacitors, Electrical and electronic engineering

## Abstract

In this study, we show that the discharge voltage pattern of a supercapacitor exhibiting fractional-order behavior from the same initial steady-state voltage into a constant resistor is dependent on the past charging voltage profile. The charging voltage was designed to follow a power-law function, i.e. $v_c(t)=V_{cc} \left( {t}/{t_{ss}}\right) ^p \;(0<t \leqslant t_{ss})$, in which $t_{ss}$ (charging time duration between zero voltage to the terminal voltage $V_{cc}$) and *p* ($0<p<1$) act as two variable parameters. We used this history-dependence of the dynamic behavior of the device to uniquely retrieve information pre-coded in the charging waveform pattern. Furthermore, we provide an analytical model based on fractional calculus that explains phenomenologically the information storage mechanism. The use of this intrinsic material memory effect may lead to new types of methods for information storage and retrieval.

## Introduction

**Figure 1:**
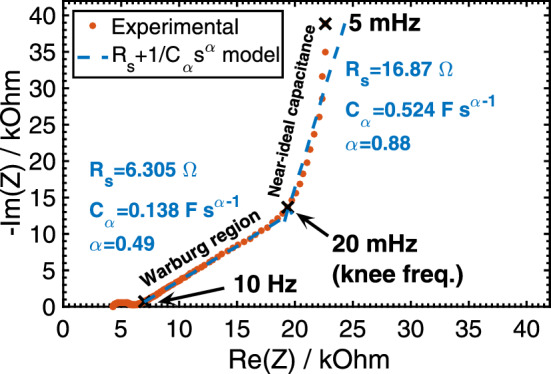
Nyquist plane representation of open-circuit spectral impedance of a NEC/TOKIN supercapacitor (part #FGR0H105ZF, rated 5.5 V, 1 F). Complex nonlinear least-squares fitting to $Z(s)=R_s+1/C_{\alpha }s^{\alpha }$ ($s=\mathrm {j}\omega$) shows two straight line regions giving the values $(R_s;C_{\alpha };\alpha )=(6.306\;\Omega ;\;0.138\;\text {F\,s}^{\alpha -1};\;0.49)$ from 10 Hz to 20 mHz, and $(16.87\;\Omega ;\;0.524\;\text {F\,s}^{\alpha -1};\;0.88)$ from 20 mHz to 5 mHz. 20 mHz is a critical frequency separating the near-ideal capacitive behavior from the Warburg region.

Many systems and processes in nature exhibit fractional-order behavior, such as vestibulo-ocular reflex^1^, neuronal activity^2^, ion channel gating^3^, viscoelasticity^4^, and disordered semiconductors^5^, which are models known to capture the existing short/long-term memory effects in these systems^6–9^. Supercapacitors are well-established types of electrochemical capacitive energy storage devices, but are also known to exhibit non-ideal, time-fractional-order electric behavior when charged by a power supply and discharged into a load^10–16^. Their electric impedance can be modeled as a series resistance ($R_s$) with a fractional-order capacitor (also known as constant-phase element, CPE^11,13,17,18^) over a certain frequency range; the reduced impedance of such a model is $Z^*(u)=1+ {1}/{(\mathrm {j} u)^{\alpha }}$ with $u=\omega (R_sC_{\alpha })^{1/\alpha }$, $C_{\alpha }$ is a fractional-order capacitance in units of F s$^{\alpha -1}$, $\alpha$ ($0<\alpha < 1$) is a dimensionless fractional exponent, and $\omega$ is the applied angular frequency in units of s$^{-1}$ (see Nyquist plot of a commercially-available NEC/TOKIN supercapacitor in Fig. 1). It is also possible to describe the impedance response over wider frequency range using more complex models such as double-dispersion Cole-Cole, Cole-Davidson, or Havriliak-Negami models ^16,19,20^. In the time-domain, the current-voltage relationship of an $R_s$-CPE-equivalent supercapacitor is expressed by the fractional-order differential equation^21,22^:

1 $$\begin{aligned} R_s C_{\alpha } \frac{d^{\alpha }V_{C_{\alpha }}(t)}{dt^{\alpha }} + V_{C_{\alpha }}(t) = v_c(t) \end{aligned}$$

where $V_{C_{\alpha }}$ is the voltage across the CPE part ($V_{C_{\alpha }}=0$ for $t\le 0$), $C_{\alpha } {d^{\alpha }V_{C_{\alpha }}(t)}/{dt^{\alpha }}$ is the current flowing through the CPE, and $v_c(t)$ is the applied charging voltage. The non-integer order differentiation in Eq. (1) is defined as^23^:

2 $$\begin{aligned} \frac{d^{-\alpha } {V}(t)}{dt^{-\alpha }} = \frac{1}{\Gamma (\alpha )} \int \limits _0^t {V}(\tau ) (t-\tau )^{\alpha -1} d\tau , \end{aligned}$$

which can be viewed as a convolution of the function V(t) with a hyperbolic function of frequency, and therefore contains a memory that progressively increases as the fractional order $\alpha$ decreases^24–26^. The physical interpretation of such fractional-order electric behavior of supercapacitors is still under debate, nonetheless, it has been widely attributed to the surface chemistry and morphological structure of the electrodes, which are usually composed of high-surface area and porous materials separated by an ionic conductor^27–30^.

Despite the fact that fractional-order models involve hereditary effects, very few robust experimental proofs of this memory have been reported and investigated^24,25,31–34^. The authors showed in a recent study that discharging a supercapacitor into a constant resistor from the same voltage-charge point reached using two different charging waveforms (step voltage and linear voltage ramp) of two different durations leads to two different responses, mostly in the short term, transient regime^33^. This indicates that knowledge of the charging pre-history matters in the determination of the current response of the device^33^. In a subsequent study, we provided an estimate of the memory effect using thee memory trace interpretation of fractional-order dynamics^34^. This paper illustrates the usage of the memory effect in supercapacitors by sequentially encoding information into the charging pattern of the device, and then uniquely retrieving this code from the discharge pattern. We investigated this effect experimentally and mathematically using fractional-order models, which means that we are leaving the physical interpretation to another study. The results are obtained on a state-of-the-art, commercially-available device offering excellent stability, and relatively high-voltage ratings which facilitates repeatability and precise measurements. However, the rated capacitance of the used device is relatively high (1 F at dc) which results in long read/write cycles. In principle, faster read/write capabilities can be achieved using fractional-order devices with lower ratings ^17,35^.

## Methods

We applied different charging voltage waveforms to a NEC/TOKIN supercapacitor, part #FGR0H105ZF, rated 5.5 V, 1 F. The device consists of six 0.917 V-aqueous electrolytic cells stacked in series; each cell is composed of two symmetric activated carbon + dilute sulfuric acid electrodes separated by a porous organic film. The voltage inputs are designed following the power-law function:

3 $$\begin{aligned} v_c(t)=V_{cc} \left( \frac{t}{t_{ss}}\right) ^p \quad (0<t \leqslant t_{ss}) \end{aligned}$$

where *p* is an exponent taking values between 0 (step voltage) and 1 (linear ramp), and $t_{ss}$ is the rise time from 0 V to the steady-state value $V_{cc}$ ($V_{cc}=5.5\;\text {V}$ for this device), after which the charging voltage is turned off. The rise time $t_{ss}$ is pre-defined so that the device will operate either in the capacitive tail from 20 mHz to 5 mHz, or in the Warburg region from 10 Hz to 20 mHz (see the two quasi-linear regions in Fig. 1). Prior to each applied charging voltage waveform, the supercapacitor was fully discharged into a constant 100 $\Omega$ resistor ($R_p$) until its voltage was equal to 1 mV. Depending on the values of *p* and $t_{ss}$, the charge waveforms used for information storage and the discharge waveforms used for information retrieval are given by the letter/number codes in Table 1. All charging/discharging experiments of the supercapacitor were programmed and executed sequentially on a Bio-logic VSP-300 electrochemical station using the EC-Lab control software. The time step for collecting data in all measurements was set to 10 ms.

**Table 1:** Table of codes illustrating encoding and decoding waveforms symbols in the form of different values of *p* and $t_{ss}$ in $v_c(t)=V_{cc} ({t}/{t_{ss}})^p$. We will be using the superscripts “*c*” and “*d*” for charge and discharge, respectively. For example $A^c_{10}$ refers to the code $A_{10}$ during charge while $A^d_{10}$ refers to the same code during discharge.

*p* $\backslash$ $t_{ss}$	550 s	275 s	110 s	55 s	27 s
1.0	$A _{10}$	$B _{10}$	$C _{10}$	$D _{10}$	$E _{10}$
0.7	$A _{07}$	$B _{07}$	$C _{07}$	$D _{07}$	$E _{07}$
0.4	$A _{04}$	$B _{04}$	$C _{04}$	$D _{04}$	$E _{04}$
0.2	$A _{02}$	$B _{02}$	$C _{02}$	$D _{02}$	$E _{02}$
0.1	$A _{01}$	$B _{01}$	$C _{01}$	$D _{01}$	$E _{01}$

## Results

**Figure 2:**
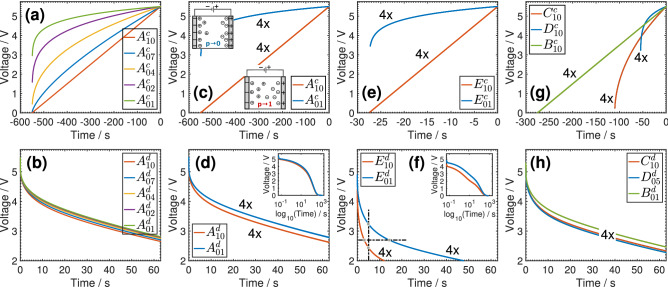
Charging sequence (first row) using a power supply with a voltage $v_c(t)=V_{cc} \left( {t}/{t_{ss}}\right) ^{p}$, and discharging sequence (second row) into a constant 100 $\Omega$ resistor of an NEC/TOKIN supercapacitor. (**a**),(**b**) depict the charge/discharge voltage patterns for different values of *p* (1.0, 0.7, 0.4, 0.2 and 0.1) and $t_{ss}=550$ s (Table 1), i.e. $A^c_{10}$/$A^d_{10}$-$A^c_{07}$/$A^d_{07}$-$A^c_{04}$/$A^d_{04}$-$A^c_{02}$/$A^d_{02}$-$A^c_{01}$/$A^d_{01}$. (**c**),(**d**) and (**e**),(**f**) show the repeatability of the process for four consecutive cycles (4x, superimposed on top of each other) of charge/discharge with $p=1.0$ and $p=0.1$; in (**c**),(**d**) waveforms $A^c_{10}$/$A^d_{01}$-$A^c_{10}$/$A^d_{01}$-$A^c_{10}$/$A^d_{01}$-$A^c_{10}$/$A^d_{01}$, and in (**e**),(**f**) waveforms $E^c_{10}$/$E^d_{01}$-$E^c_{10}$/$E^d_{01}$-$E^c_{10}$/$E^d_{01}$-$E^c_{10}$/$E^d_{01}$. In (**g**),(**h**), we applied the sequence of charge/discharge codes [$B^c_{10}$/$B^d_{10}$- $C^c_{05}$/$C^d_{05}$- $D^c_{01}$/$D^d_{01}$]–[$B^c_{10}$/$B^d_{10}$- $C^c_{05}$/$C^d_{05}$- $D^c_{01}$/$D^d_{01}$]–[$C^c_{05}$/$C^d_{05}$- $D^c_{01}$/$D^d_{01}$- $B^c_{10}$/$B^d_{10}$]–[$C^c_{05}$/$C^d_{05}$- $D^c_{01}$/$D^d_{01}$- $B^c_{10}$/$B^d_{10}$] using three values of $t_{ss}$ and three values of *p* (Table 1) demonstrating the possibility of two-dimensional encoding as well as the repeatability of the process ($4\times$).

The experimental results are shown in Fig. 2. In the first row of the figure, we show several combinations of the charging voltage waveforms applied to the supercapacitor by varying the values of *p* and $t_{ss}$ (see Table 1). The time scale is shifted by $-t_{ss}$ to represent past events. The second row of the figure shows the first 60 s of the resulting voltage discharge waveforms into the same 100 $\Omega$ resistor. Here the potentiostat acts as a constant resistance by controlling the current to maintain the ratio voltage/current constant. Fig. 2b shows five different voltage discharge profiles ($A_{10}^d$, $A_{07}^d$, $A_{04}^d$, $A_{02}^d$, $A_{01}^d$) after the device was charged with five different voltage waveforms ($A_{10}^c$, $A_{07}^c$, $A_{04}^c$, $A_{02}^c$, $A_{01}^c$) (Fig. 2a). The value of $t_{ss}$ is made long enough so that the device will operate within its low frequency capacitive tail (Fig. 1). All discharging waveforms show first a quick voltage drop of *ca.* 0.5 V from the initial voltage $V_{cc}=5.5\;\text {V}$ into the internal series resistance of the device ($R_s$), followed by a non-Debye inverse power law profile (i.e. $v_d(t) \propto t^{-\alpha }$^24,25,36,37^). In Fig. 2d, we show the discharge profiles of the device for four consecutive cycles ($4\times$) alternating between code $A_{10}^c$ and code $A_{01}^c$ (Fig. 2c). The corresponding discharge waveform codes $A_{10}^d$ and $A_{01}^d$ are perfectly superimposed on each other demonstrating the repeatability of the charge/discharge process and the good stability of the device between one sequence to another. The results in Fig. 2a–d show that the supercapacitor voltage discharge profile into the same constant resistance depends on the exponent *p* in the charging voltage profile, and thus on its prehistory. This would not be the case for an ideal capacitor for which the exponential decaying voltage $v_d(t) = V_{cc} \exp \left( -{t}/{R_pC}\right)$ depends on the initial voltage $V_{cc}$, but not on how this voltage has been reached. In other words, information can be encoded in the exponent *p* being for example 1.0 or 0.1 (Fig. 2c,d) which can be used to encode a binary data sequence, or in multi-level logic using multiple values of *p* (Fig. 2a,b).

The effect of the parameter $t_{ss}$ is examined in Fig. 2e,f, which depict the voltage profiles that were obtained in a similar way to the results shown in Fig. 2c,d, respectively, but with a faster charging rate ($t_{ss}=27\,\text {s}$). This value of $t_{ss}$ corresponds to about 37 mHz frequency which belongs to the Warburg region and not the capacitive tail anymore (see Fig. 1). It is clear from Fig. 2f that the superposition of the code plots ($E_{10}^d$ and $E_{01}^d$) obtained in response to the charging voltage codes $E_{10}^c$ and $E_{01}^c$ is again impeccable. Additionally, the difference between the discharge waveforms is more pronounced than when $t_{ss}$ was set to $550\,\text {s}$ (Fig. 2d) at which point the supercapacitor behaved as a near-ideal capacitor. Although the values of $R_s$ and $C_{\alpha }$ are not the same for these two cases, the correlation between the discharging and the charging voltage waveforms is stronger as $\alpha$ decreases (Eqs. 1 and 2) which makes $t_{ss}$ another possible information coding dimension.

As for the decoding of the discharge pattern corresponding to a specific charge pattern, this can be carried out by a simple adaptive thresholding method either for a fixed time or fixed voltage. For example, as shown in Fig. 2f, the thresholding can be performed at 5 s from the beginning of the discharge giving the two voltage values of 2.441 and 3.238 V for $p=1.0$ and $p=0.1$ (i.e. a difference of 0.797 V), respectively. Alternatively, it can be performed at a fixed voltage value of 2.7 V for example, leading to decoded time intervals of 3.109 and 14.31 s for $p=1.0$ and $p=0.1$, respectively. It is important to note that decoding from the discharging waveform should be carried out way before the device is fully discharged, as shown in decimal algorithm scale in the insets in Figs. 2d,f depicting quick convergence of the voltage-time profiles. The reason is that fractional-order behavior is space- and time-dependent; i.e. only in the transient time does the order of the state-space (represented by the values of $\alpha$) manifest itself leading to the memory effect^33,34^. In the steady-state time, fractional-order behavior becomes space-order independent.

Another set of experimental results are shown in Fig. 2g,h, in which charge/discharge of the device followed the sequence [$B_{10}$-$C_{05}$-$D_{01}$]–[$B_{10}$-$C_{05}$-$D_{01}$]–[$C_{05}$-$D_{01}$-$B_{10}$]–[$C_{05}$-$D_{01}$-$B_{10}$]. This indicates the possibility of two-dimensional memory encoding using *p* and $t_{ss}$.

## Discussion

The memory effect can be explained analytically as follows. The voltage discharge response of the supercapacitor, modeled as an $R_s$-CPE equivalent circuit, into a parallel resistance $R_p$ is given by^38^:

4 $$\begin{aligned} v_d(t) = v_d(0) \frac{R_p}{R_p+R_s} \text {E}_{\alpha ,1}\left( - \frac{t^{\alpha }}{\tau _p^{\alpha }+\tau _s^{\alpha }} \right) \end{aligned}$$

where $\tau _p=\left( R_p C_{\alpha } \right) ^{1/\alpha }$, $\tau _s=\left( R_s C_{\alpha } \right) ^{1/\alpha }$, $\text {E}_{\alpha ,\beta }(z)= \sum _{k=0}^\infty z^k/\Gamma (\alpha \,k+\beta )$ with ($\alpha >0$, $\beta >0$) is the two-parameter Mittag-Leffler function, and the initial voltage $v_d(0)$ is given by $v_d(0) = V_{cc} - R_s i_c(t_{ss})$ where $i_c(t)$ is the charging current obtained as $dq_c(t)/dt$ (see Eq. 7). For $R_p \gg R_s$, Eq. (4) can be rewritten in a dimensionless form as follows:

5 $$\begin{aligned} {\tilde{v}}_d{({\tilde{t}}_p)} \simeq R_{(p,m,\alpha )} \text {E}_{\alpha ,1} \left( - {\tilde{t}}_p^{\,\alpha } \right) \end{aligned}$$

where ${\tilde{v}}_d{({\tilde{t}}_p)}=v_d(t)/V_{cc}$, ${\tilde{t}}_p =(t/\tau _p)$ and $m=(t_{ss}/\tau _s)$. The Mittag-Leffler function in Eq. (5) depends on the CPE parameters $\alpha$ and $C_{\alpha }$ (and on $R_p$), whereas the parametric function $R_{(p,m,\alpha )}$ is dependent also on how the device has been charged via the selection of one or both of the applied waveform parameters *p* and $t_{ss}$. For an ideal capacitor, i.e. with $\alpha =1$ and $R_s=0$, we verify that Eq. (5) simplifies to ${\tilde{v}}_d{({\tilde{t}}_p)} = \exp (- {\tilde{t}}_p)$ which does not depend on its charge prehistory, as expected.

**Figure 3:**
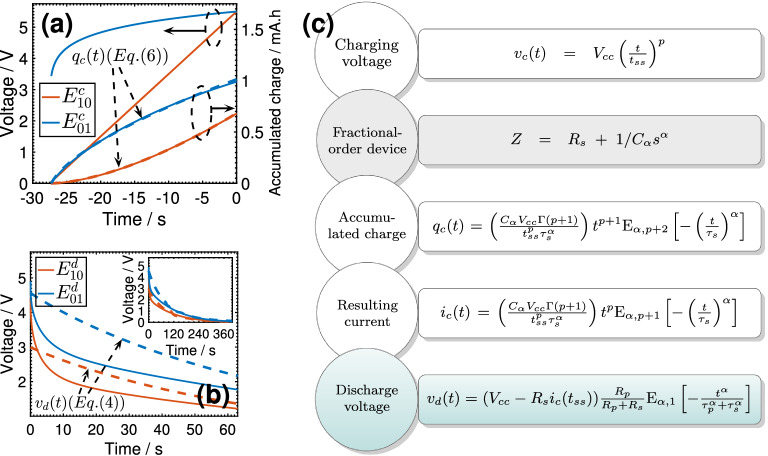
(**a**) Supercapacitor charging using the voltage waveforms $v_c(t)=V_{cc} \left( {t}/{t_{ss}}\right) ^{p}$ with $t_{ss}=27 \text {s}$ and $p=1.0$ and 0.1 (i.e. $E_{10}^c$ and $E_{01}^c$) and the resulting time-charge profiles. (b) Illustrates the corresponding discharging voltage waveforms (i.e. $E_{10}^d$ and $E_{01}^d$). Experimental measurements are plotted in solid lines and simulations are represented by dashed lines. (**c**) Flowchart representing the process and equations of the electric variables during charge (i.e. applied voltage, accumulated charge, and corresponding current), and voltage during discharge into a constant resistor (the device is considered. to be a fractional-order supercapacitor of impedance $Z=R_s+1/C_{\alpha }s^{\alpha }$ and being charged with a voltage function $v_c(t)=V_{cc} \left( {t}/{t_{ss}}\right) ^p$).

Now, to show the memory relationship, we derive the expression for the electrical charge stored in the device subjected to the charging waveform $v_c(t) = V_{cc} \left( {t}/{t_{ss}}\right) ^p$ as follows^33^:

6 $$\begin{aligned} q_c(t) = \left( \frac{C_{\alpha } V_{cc} \Gamma (p+1)}{ t_{ss}^p \tau _s^{\alpha } } \right) t^{p+1} \text {E}_{\alpha ,p+2} \left[ - \left( \frac{t}{\tau _s} \right) ^{\alpha } \right] \vspace{.1cm} \end{aligned}$$

from which the current is found to be:

7 $$\begin{aligned} i_c(t) = \left( \frac{C_{\alpha } V_{cc} \Gamma (p+1)}{ t_{ss}^p \tau _s^{\alpha } } \right) t^{p} \text {E}_{\alpha ,p+1} \left[ - \left( \frac{t}{\tau _s} \right) ^{\alpha } \right] \vspace{.1cm} \end{aligned}$$

[Note that Eq. (6) is obtained by equating $i(t) = dq/dt$ with $C_{\alpha } {d^{\alpha }V_{C_{\alpha }}(t)}/{dt^{\alpha }}$ where $V_{C_{\alpha }}(t)$ is the solution of the fractional-order differential equation $V_{cc} \left( {t}/{t_{ss}}\right) ^p = V_{C_{\alpha }} + R_s C_{\alpha } {d^{\alpha }V_{C_{\alpha }}(t)}/{dt^{\alpha }}$ obtained using the inverse Laplace transform identity ${\mathscr {L}}^{-1} \left( \frac{k! s^{\alpha -\beta }}{(s^{\alpha } + \lambda )^{k+1} } \right) = t^{\alpha \,k +\beta -1} E_{\alpha ,\beta }^{(k)} [ - \lambda t^{\alpha } ]$ with ($k=0$, $\beta =p+1$, $\lambda =\tau _s^{-\alpha }$)^23^]. At $t=t_{ss}$, the steady-state charge $q_c(t_{ss})$ is a function of both parameters of the charge waveform (*p* and $t_{ss}$) and those of the supercapacitor. [Equation (6) simplifies to $q_c(t) = CV_{cc}t/t_{ss}$ for an ideal capacitance ($C_{\alpha }=C$ and $\alpha =1$) using the identity $E_{1,3}(z) = (\exp (z)-z-1)/z^2$ which applies when $p=1$. If $p = 0$ (step voltage), the charge $q_c(t)=CV_{cc}$, otherwise $q_c(t)$ is a function of *p*]. This is in line with our recent findings in which we highlighted that charging a supercapacitor with a voltage input results in a device and waveform-dependent accumulated electric charge^12,33,34^. In a dimensionless form, Eq. (6) looks like this:

8 $$\begin{aligned} {\tilde{q}}_c({\tilde{t}}_s)= S_{(p,m,\alpha )} {\tilde{t}}_s^{\,p+1} \text {E}_{\alpha ,p+2} \left( -{\tilde{t}}_s^{\,\alpha } \right) \end{aligned}$$

where ${\tilde{t}}_s=t/\tau _s$. In Fig. 3a, we show the measured and simulated (using Eq. 6) charge function $q_c(t)$ for a fixed value of $t_{ss}$ equal to 27 s, and steady-value voltage of 5.5 V. Two values of the parameter *p* (i.e. 1.0 and 0.1) are selected. Equation (6) is in excellent agreement with the experiment using the values ($R_s, C_{\alpha }, \alpha$) equal to ($10\,\Omega , 35\,\text {mF\,s}^{\alpha -1}, 0.48$) for $p=1.0$, and equal to ($10\,\Omega , 36\,\text {mF\,s}^{\alpha -1}, 0.49$) for $p=0.1$ obtained using least-squares fitting. Experimentally, the accumulated charge at time $t_{ss}$ is found to be 0.68 and 0.99 mA h$^{-1}$ for $p=1.0$ and 0.1, respectively. Given that all parameters in Eq. (6) are fixed apart from *p*, it is evident that the information encoded in the value of *p* is stored as $q_c(t)$. In Fig. 3b, we show the resulting discharge voltage along with simulation using Eq. (4). The best fit is found using the values ($R_s, C_{\alpha }, \alpha$) equal to ($10\,\Omega , 803\,\text {mF\,s}^{\alpha -1}, 0.96$) for $p=1.0$, and equal to ($14.24\,\Omega , 855\,\text {mF\,s}^{\alpha -1}, 0.95$) for $p=0.1$, which can be improved if a sliding mode fitting is adopted instead. Note that the increase of the values of $\alpha$ and $C_{\alpha }$ towards those of a near-ideal capacitance is the result of the slow discharge rate.

## Conclusion

We have shown that the voltage discharge of a supercapacitor that exhibits fractional-order temporal dynamics depends uniquely on the way by which it was charged. Figure 3c recapitulates the process from charging the device by an external power supply, to the accumulation of charge and the creation of current, and their relationship with the discharge voltage function which depends on the voltage charging parameters *p* and $t_{ss}$. In other words, the supercapacitor “remembers” the pattern by which it was charged, and as a result, discharges accordingly.

The experimental results reported here were obtained on a commercial, high-capacitance supercapacitor (designed for the purpose of energy storage) which resulted in slow information storage and retrieval. Higher read-write rates should be possible by using low-capacitance, fractional-order devices^35^. In Fig. S1 we show simulation results of the effect of lowering the value of $C_{\alpha }$ (keeping all other parameters unchanged, i.e. $\alpha =0.95$, $R_s=14.42\,$Ohm, $R_p=100\,$Ohm) on the discharging voltage-time profiles illustrating faster read time. However, it is important to ensure stability over a large number of cycles, as well as to reduce the effect of any parasitic capacitance for properly assessing the memory effect from low-capacitance devices.

## Supplementary information

Supplementary information.
