# Supplementary material for: Possibility of information encoding/decoding using the memory effect in fractional-order capacitive devices
Source: Sci Rep. 2021 Jun 25;11:13306. doi: 10.1038/s41598-021-92568-3 (PMC8233438; doi:10.1038/s41598-021-92568-3)
Supplement: Supplementary file 1 — Supplementary information. [file 41598_2021_92568_MOESM1_ESM.pdf]

Possibility of Information Encoding/Decoding using the Memory Effect in Fractional-order Capacitive Devices

Anis Allagui[1,2,4,*] **and Ahmed S. Elwakil**[3,5,6]

[1]Dept. of Sustainable and Renewable Energy Engineering, University of Sharjah, PO Box 27272, Sharjah, United Arab Emirates
[2]Research Institute of Sciences and Engineering, University of Sharjah, PO Box 27272, Sharjah, United Arab Emirates
[3]Dept. of Electrical Engineering, University of Sharjah, PO Box 27272, Sharjah, United Arab Emirates
[4]Dept. of Mechanical and Materials Engineering, Florida International University, Miami, FL33174, United States
[5]Nanoelectronics Integrated Systems Center, Nile University, Cairo 12588, Egypt
[6]Dept. of Electrical and Computer Engineering, University of Calgary, Calgary, Alberta T2N 1N4, Canada
[*]Correspondent author: aallagui@sharjah.ac.ae

SUPPORTING INFORMATION

From Eq. 4 in the manuscript, i.e.

$$v_d(t) = v_d(0)\frac{R_p}{R_p+R_s}\mathrm{E}_{\alpha,1}\left(-\frac{t^\alpha}{\tau_p^\alpha+\tau_s^\alpha}\right)$$

it is clear that the read operation (discharge voltage) is proportional to the Mittag-Leffler function $\mathrm{E}_{\alpha,1}(x)$. In Fig. S1 we show the effect of lowering the values of C_α on $v_d(t)$ keeping all other parameters as given in the manuscript (see figure caption). Recall that $\tau_p = (R_pC_\alpha)^{1/\alpha}$, $\tau_s = (R_sC_\alpha)^{1/\alpha}$. It is clear from the figure that faster reading times can be achieved when devices of lower C_α values are used.

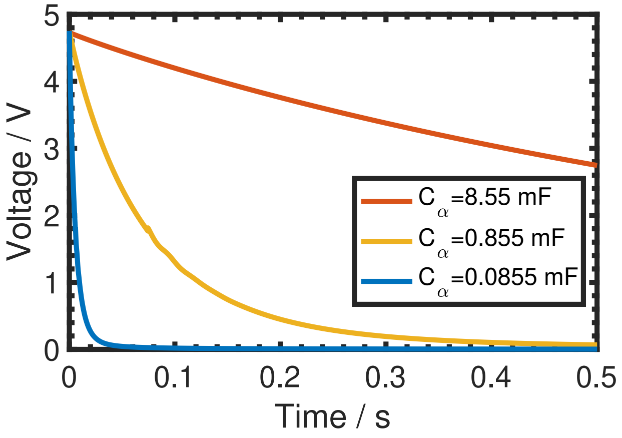

Figure S1. Plot of discharging voltage waveforms $v_d(t)$ using $\alpha = 0.95$, $R_s = 14.42\,\mathrm{Ohm}$, $R_p = 100\,\mathrm{Ohm}$ (see Fig. 3(b) in the manuscript) for different values of C_α as indicated in the legend
